# Optimizing the Sensor Placement for Foot Plantar Center of Pressure without Prior Knowledge Using Deep Reinforcement Learning

**DOI:** 10.3390/s20195588

**Published:** 2020-09-29

**Authors:** Cheng-Wu Lin, Shanq-Jang Ruan, Wei-Chun Hsu, Ya-Wen Tu, Shao-Li Han

**Affiliations:** 1 Department of Electronic and Computer Engineering, National Taiwan University of Science and Technology, Taipei 106, Taiwan; M10702129@mail.ntust.edu.tw (C.-W.L.); sjruan@mail.ntust.edu.tw (S.-J.R.); 2 Graduate Institute of Biomedical Engineering, National Taiwan University of Science and Technology, Taipei 106, Taiwan; wchsu@mail.ntust.edu.tw; 3 Sijhih Cathay General Hospital, New Taipei 221, Taiwan; cgh04234@cgh.org.tw

**Keywords:** plantar pressure, center of pressure, sensor placement optimization, deep reinforcement learning, soft actor–critic discrete

## Abstract

We study the foot plantar sensor placement by a deep reinforcement learning algorithm without using any prior knowledge of the foot anatomical area. To apply a reinforcement learning algorithm, we propose a sensor placement environment and reward system that aims to optimize fitting the center of pressure (COP) trajectory during the self-selected speed running task. In this environment, the agent considers placing eight sensors within a 7 × 20 grid coordinate system, and then the final pattern becomes the result of sensor placement. Our results show that this method (1) can generate a sensor placement, which has a low mean square error in fitting ground truth COP trajectory, and (2) robustly discovers the optimal sensor placement in a large number of combinations, which is more than 116 quadrillion. This method is also feasible for solving different tasks, regardless of the self-selected speed running task.

## 1. Introduction

The definition of foot plantar pressure is the distribution of force between the foot’s sole and the support surface. Plantar pressure measurement systems have been used in several applications, such as sports performance analysis and injury prevention [1], gait monitoring [2], and biometrics [3]. In  the literature, various sensor placement patterns, based on modified from the foot anatomical area or filled with mesh-like sensors array, were discussed by Razak et al. [4]. The design approach filled with mesh-like sensors increases the measuring accuracy but also increases the prices. However, reducing the number of sensors and achieving acceptable accuracy is challenging. Usually, finding a sensor placement pattern is determined by a human expert. By contrast, this paper proposes another new design approach for plantar sensor placement based on plantar pressure data and deep reinforcement learning (DRL) [5,6] algorithm. This approach uses the center of pressure (COP) trajectory to evaluate the sensor placement quality. Using this mechanism, we are trying to find new placement patterns that human knowledge has not yet discovered.

Reinforcement learning (RL) [7] is an algorithm that consists of an environment and an agent and trains the agent’s policy through feedback from the environment. In many complex domains, reinforcement learning is the only feasible way to train a program to perform at high levels. Furthermore, deep reinforcement learning (DRL) merges deep learning (DL) [8] and reinforcement learning (RL) algorithms. Deep learning is a branch of machine learning which uses an artificial neural network to extract information from high dimensional data and has led to breakthroughs in computer vision [9,10,11,12] and speech recognition [13,14]. Within DRL, it uses a deep neural network as a function approximator [15], which not only allows this algorithm to extract information from high dimensional data but also scale-up the ability to solve more complex problems. Moreover, DRL has accomplished many achievements, such as mastering the game of go without human knowledge [16] and winning the world champions in a multiplayer real-time strategy game [17], in modern machine learning. For the sensor placement problem, many combinations for sensor placement solving by brute force are not feasible, so we adopted a DRL to this problem.

We have organized the rest of this paper in the following way: first, we describe the data collection for self-selected speed running plantar pressure videos and the preprocessing. Then, we propose the environment and the reward system for designing the sensor placement. This environment and reward system aim to optimize the sensor placement for COP accuracy and adapt to DRL. Third, we briefly illustrate the Soft Actor–Critic Discrete (SAC-Discrete) [18], a discrete version of the Soft Actor–Critic (SAC) RL algorithm [19], and apply it for the sensor placement task with some simple testing data, which we created. Fourth, we utilize the Population-Based Training (PBT) [20] method to tune the hyperparameter using SAC-Discrete. Applying this method enhances training stability and performance in our sensor placement task. Finally, we feed the plantar pressure videos to the sensor placement environment and then present the results and conclusions.

## 2. Materials and Methods

### 2.1. Collecting Plantar Pressure Video

#### 2.1.1. Participants and Experimental Protocol

Fifteen subjects (all male, age: 23.63±2.15 years, mass: 72.49±3.16 kg, height: 175.49±5.73 cm, Body Mass Index: 21.47±2.18) volunteered to participate in the study, and they are all healthy and no known lower limb injuries.

#### 2.1.2. Experimental Protocol

Each subject needs to run for three minutes with their self-selected speed on the treadmill. The data logger is triggered by an external trigger button when a subject is comfortable with the treadmill’s current speed. Furthermore, they are wearing the same model of shoes with their proper size.

#### 2.1.3. Self Selected Speed Plantar Pressure Video Collection

**Figure 1:**
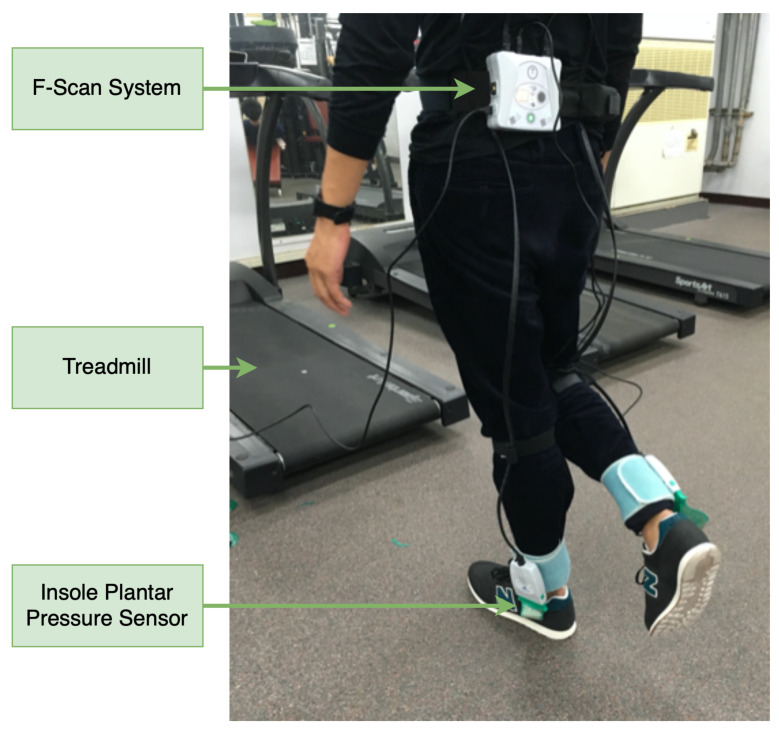
Illustration of a self-selected speed plantar pressure video collection experiment.

Plantar pressure video is recorded with the F-Scan [21] system by Tekscan, which receives plantar pressure with an insole pressure sensor array. This system contains a pair of resistive sensor [22] sheets placed on top of the insole and applying double-sided tape to avoid sensor sheets slipping during recording. The pressure range of this sensor sheet for this experiment is 1–150 psi (approximately 7–1000 kPa). The F-Scan system’s recording software version is 7.50-07, and, before recording, the sensor sheet is calibrated by this software. The maximum spatial resolution of this F-Scan hardware/software system is up to 750 Hz. In this experiment, we set the acquisition frequency at 100 Hz, so the recording video’s output frame rate is also at 100 Hz. Since the plantar pressure video is obtained from the F-Scan software, which has done the calibration, this video’s spatial resolution is 21 × 60, and the unit of each pixel value is kPa. The F-Scan system starts to record the data when a subject is comfortable with the treadmill’s current speed and finishes a record after three minutes. This experiment is illustrated in Figure 1.

#### 2.1.4. Data Preprocessing

Plantar pressure videos collected from the F-Scan system are preprocessed to construct a data set; for each episode, the sensor placement environment will randomly select a plantar pressure video within this data set to calculate rewards. The preprocessing steps are as follows:

1. A gait cycle consists of the stance phase and the swing phase; during the swing phase, the F-Scan system will not receive any pressure information. Thus, we remove the swing phase within a three-minute plantar pressure video by splitting it into many stance-phase plantar pressure videos.
2. To reduce the amount of stance-phase plantar pressure videos, we divide those videos into five equal groups with the time sequence and randomly choose one video from each group.
3. The stance-phase plantar pressure videos are cropped to remove the white border, which is a row or column that does not receive any pressure within this plantar pressure video.
4. After cropping the stance-phase plantar pressure videos, each video presents different spatial resolution. Thus, we downsample each video to 7 × 20 by the pressure formula P=F/A.

**Figure 2:**
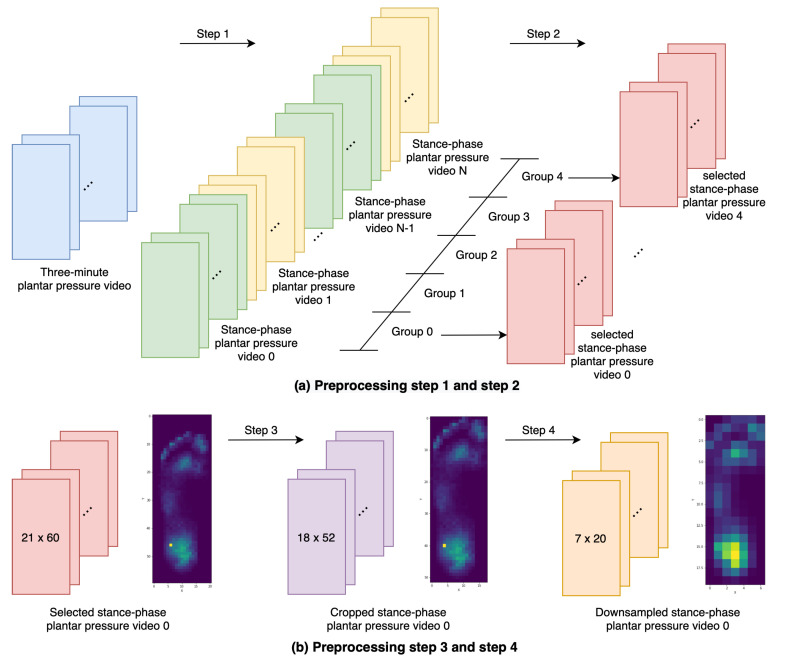
Illustration of preprocessing steps. (**a**) The green and yellow videos represent the stance-phase plantar pressure video; each video’s total frame count depends on its stance-phase duration. The pink videos represent the stance-phase plantar pressure video that randomly selected from five equal groups. (**b**) In step three and step four, we use one of the chosen videos for a demonstration; the image beside each video is its pixel-wise accumulated image, which utilizes to visual the cropping and resampling processes. The purple video represents the cropped video, and the orange video is the final result, which has a 7 × 20 spatial resolution.

For each subject, this experiment collects two three-minute plantar pressure videos; one is the left foot, and the other is the right foot. After the preprocessing, data collected from a subject produces ten 7 × 20 stance-phase videos, as Figure 2 shows. Since fifteen subjects join this experiment, there are 150 stance-phase plantar pressure videos using in the sensor placement environment.

### 2.2. Sensor Placement Environment and Reward System

Reinforcement learning is an algorithm that consists of an agent and an environment. For each time step, the environment provides the state’s information for the agent and then the agent using it to select an action. After the action has taken, the environment updates its state and then offers the next state’s information and reward to the agent. These interactions between the environment and the agent produce a serial of state–action pairs. The length of this state–action pairs depends on the environment’s termination condition and could also be infinite. By using those state–action pairs, the RL algorithm reinforces the agent’s policy to maximize the environment’s accumulative reward. To optimize the sensor placement for the COP trajectory during the self-selected speed running task, we present a sensor placement environment and a reward system.

#### 2.2.1. Sensor Placement Environment

**Figure 3:**
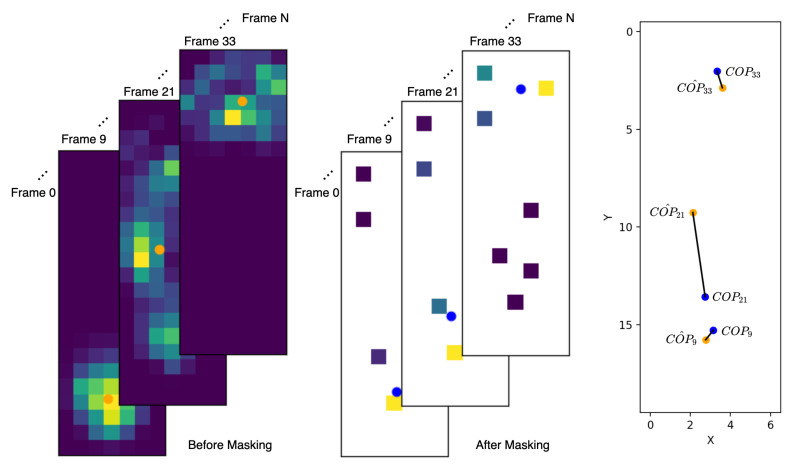
The distance between masked and non-masked COP positions. For demonstration, we chose three frames in this plantar pressure video, the orange dot is the non-masked COP position and the blue dot is the masked COP position. The black line is the distance between these two COP positions at each frame.

**Figure 4:**
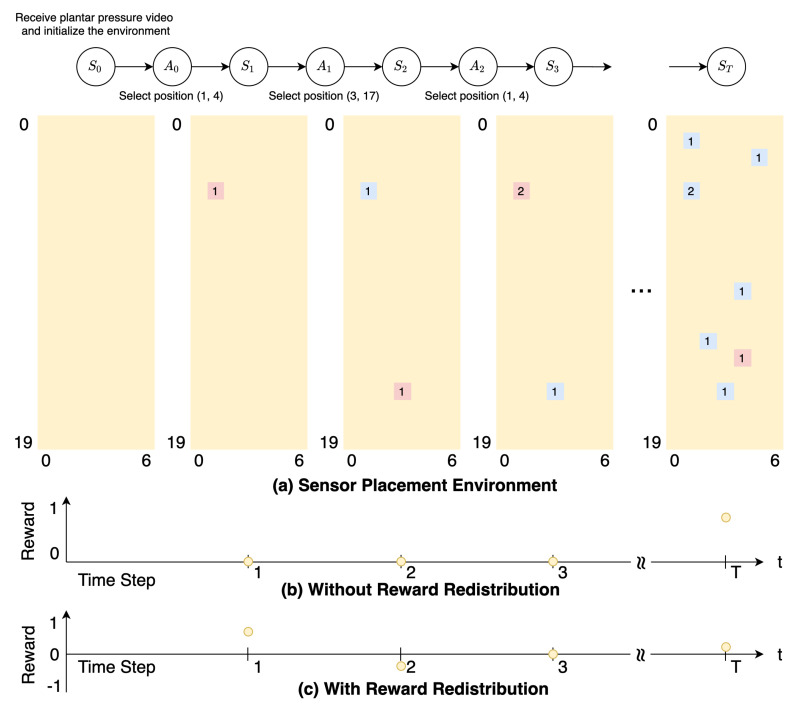
Schematic illustration of the sensor placement environment and reward system. (**a**) The pink point and the blue point represent the agent’s current and previous selecting position separately, and the number represents the sensors count on this position. The notation $S_{T}$ represents the terminate state. $S_{3}$ demonstrates the situation when the agent selects a position where another sensor already existed. (**b**) Due to the reward and the next state provided by the environment simultaneously, the first reward starts at $S_{1}$. Without reward redistribution, the agent only receives the reward in the terminate state. (**c**) The redistributed reward is the current and previous accumulative reward difference. Since $S_{3}$ has the same masked position as $S_{2}$, they get the same accumulative reward in this episode; the redistributed reward at $S_{3}$ is zero. It is also possible to get a negative reward, as shown at $S_{2}$.

At the initial state, the sensor placement environment gets a plantar pressure video, which will be used to calculate the reward, and provides an empty 7 × 20 board information to the agent. Figure 3 shows the plantar pressure video. The agent owns eight sensors at the beginning, which can be placed on this empty board. For each time step, the agent places one of its sensors on the board. It does not matter whether another sensor is placed on a position where other sensors already exist; in other words, having multiple sensors on the same position is allowed. The terminating condition for the sensor placement environment is that the agent finishes placing all of its sensors. When this episode terminates, the agent will receive the only reward given by the environment; it means that the agent will only receive a zero as the reward until it reaches the terminate state. The agent’s main objective is to place the sensors in the crucial positions to get the maximum reward at the end of this episode. Figure 4a illustrates the interaction between the agent and the environment, and Figure 4b shows the reward given by the environment.

#### 2.2.2. Reward System

In reinforcement learning, planning a reward system is essential. The positive reward given by the environment encourages the agent to do more actions that can receive this reward. With the sensor placement environment scenario, it encourages the agent to arrange sensor positions for fitting the COP trajectory to get a higher reward at the terminate state. The environment calculates a reward using a plantar pressure video and the final sensor positions, which the agent determines. For each episode, the environment can use different plantar pressure videos to calculate the reward. It means that, even if the agent places all of its sensors in the same positions in each episode, the reward could be changed. To calculate the reward, we first introduce the COP trajectory formula for a plantar pressure video as follows:

(1) $$COP_{n}=\left(COP_{n,x},COP_{n,y}\right)=\left(\frac{{\sum pressure}_{n}\times {coordinate}_{x}}{{\sum pressure}_{n}},\frac{\sum {pressure}_{n}\times {coordinate}_{y}}{\sum {pressure}_{n}}\right)$$

The COP trajectory is a serial of points lying on the 2D plane, and the length of this series is the video frame count. In this formula, *n* is the index for the frame number, the $pressure_{n}$ represents a pixel value within the *n*-th frame, and $coordinate_{x}$ and $coordinate_{y}$ are the relative position for that pixel. Now, we describe how to calculate the reward with the sensor positions given by the agent. The environment can use the sensor positions as a pixel-wise mask for the plantar video to get two various COP trajectories. One is the COP trajectory calculated from the original plantar pressure video; another is the COP trajectory calculated from the masked plantar pressure video. Optimizing the sensor positions for fitting the COP trajectory can achieve by minimizing the distances between COP positions calculated from the original and the masked ones for each frame, as Figure 3 shown. Thus, the reward function is defined as follows:

(2) $$\begin{array}{cc} \; & reward={\left(1-\frac{\sum_{n=0}^{N}\sqrt{{\left(COP_{n,x}-{C\hat{OP}}_{n,x}\right)}^{2}+{\left(COP_{n,y}-{C\hat{OP}}_{n,y}\right)}^{2}}}{(N+1)\times max\;distance}\right)}^{0.4}, \end{array}$$

(3) $$\begin{array}{cc} \; & max\;distance=\sqrt{{\left(video\;width\right)}^{2}+{\left(video\;height\right)}^{2}} \end{array}$$

where $\left({C\hat{OP}}_{n,x},{C\hat{OP}}_{n,y}\right)$ denotes the COP position calculated from the plantar pressure video, $\left(COP_{n,x},COP_{n,y}\right)$ denotes the masked version, and N+1 is the total frame count. The distance between two COP positions is normalized to [0,1] by divided by maxdistance. Using one minus the normalized distance as the reward, when the distance is zero, the agent will get the maximum reward, which is one. The exponent 0.4 in this equation is used to increase the precision for a smaller distance and encourages the agent to get a better score. Finally, average the reward over each frame by summing up the reward for each frame and then dividing by N+1.

#### 2.2.3. Reward Redistribution

Training an agent in a delayed rewards environment is a challenging problem in RL. First, since the agent can not immediately notice if its action is good or bad, reinforcing its policy becomes harder. Second, it also takes time to propagate the delayed rewards to the current state, which means it takes a much longer time for training. To solve this problem in the sensor placement environment, we used the concept proposed in the RUDDER algorithm [23]. RUDDER’s idea is to distribute the delayed reward to those actions that cause this delayed reward to happen and can be implemented by the following steps:

1. Using a Long Short-term Memory (LSTM) model to construct a sequence-to-sequence supervised learning task [24]. The serial of state–action pairs as the input and the delayed reward as the label. The output sequence of this model can be treated as the accumulative reward at each state.
2. After this supervised learning is finished, the redistributed rewards for each state will be calculated by differencing the current and previous state accumulative reward.
3. Replacing the original reward with the redistributed rewards then trains the agent with any feasible RL algorithm.

Since the accumulative reward in the sensor placement environment can be calculated with Equation (2) for each state, we can skip the first step. The rewards after the RUDDER algorithm are shown in Figure 4c.

### 2.3. Soft Actor–Critic Discrete

Various deep RL algorithms have been proposed in recent years, like Asynchronous Advantage Actor–Critic (A3C) [25], Proximal Policy Optimization (PPO) [26], and Soft Actor–Critic (SAC) [19]. We chose the discrete version of Soft Actor–Critic (SAC-Discrete) [18] for the following reasons: First, the SAC-Discrete objective function optimizes the agent’s policy while also maximizing its policy entropy. This objective function increases training stability and encourages the agent to discover the environment. Second, SAC-Discrete is an off-policy RL algorithm; it increases the data reusability so that it can reduce training time. In the sensor placement environment, different sensor placement patterns can get the same reward at the end of the episode, using SAC-Discrete can discover all of those patterns. In this section, we first introduce notation, followed by the maximum entropy reinforcement framework, and finally the SAC-Discrete algorithm.

#### 2.3.1. Notation

An RL problem can be mathematically formulated by a Markov Decision Process (MDP). An MDP P consists of five tuples, P=(S,A,R,p,γ), where S is a set of states *s* (random variable of $S_{t}$ at time *t*), A is a set of actions *a* (random variable of $A_{t}$ at time *t*) and R is a set of rewards *r* (random variable of $R_{t+1}$ at time *t*). P has transition-reward distributions as follows $p(S_{t+1}=s^{{}^{\prime }},R_{t+1}=r|S_{t}=s,A_{t}=a)$ conditioned on state–action pairs at time *t*. γ∈[0,1] is a discount factor that ensures an MDP will converge. We often equip an MDP P with a policy π. A given policy $\pi (a_{t}|s_{t})$, $\rho_{\pi }\left(s_{t}\right)$ denotes the state marginals of transition-reward distributions, and $\rho_{\pi }\left(s_{t},a_{t}\right)$ denotes the state–action marginals of transition-reward distributions.

#### 2.3.2. Maximum Entropy Reinforcement Framework

The maximum entropy reinforcement framework varies the standard RL objective function $\sum_{t}E_{\left(s_{t},a_{t}\right)∼\rho_{\pi }}\left[\gamma^{t}r\left(s_{t},a_{t}\right)\right]$; this framework maximizes the expected sum of rewards, while maximizing its policy entropy as the following equation:

(4) $$\pi^{*}=\underset{\pi }{\mathrm{argmax}}\sum_{t=0}^{T}E_{\left(s_{t},a_{t}\right)∼\rho_{\pi }}\left[\gamma^{t}r\left(s_{t},a_{t}\right)+\alpha H\left(\pi \left(.|s_{t}\right)\right)\right]$$

where $\pi^{*}$ is the optimal policy, *T* is the number of time steps, and $H(\pi \left(.|s_{t}\right))$ is the entropy of π at state $s_{t}$. The temperature parameter α is a hyperparameter within the equation, determining the relative importance of the entropy term versus the reward. Thus, it also can be tuned during training time; when α is close enough to 0, this equation falls back to the standard RL objective function.

To reinforce the policy in the RL algorithm is to alternate between the policy evaluation and the policy improvement. The discrete setting of soft policy iteration for the maximum entropy reinforcement framework is presented in [18]. First, the policy evaluation is as follows:

(5) $$V\left(s_{t}\right):=\pi {\left(s_{t}\right)}^{T}\left[Q\left(s_{t}\right)-\alpha \log\left(\pi \left(s_{t}\right)\right)\right]$$

In the discrete action setting, policy outputs the probability for each possible action $\pi \in {\left[0,1\right]}^{\left|A\right|}$, $Q(s_{t})$ is the soft Q-function that outputs the Q-value for each action $Q\left(s_{t}\right):S\to R^{\left|A\right|}$. $V(s_{t})$ is the state-value function defined as the dot product of the action probabilities and the Q-values with entropy turn. Then, the policy improvement is achieving by a policy gradient method [27]; its objective function is as follows:

(6) $$J_{\pi }\left(\phi \right)=E_{s_{t}∼D}\left[\pi_{\phi }{\left(s_{t}\right)}^{T}\left[\alpha \log\left(\pi_{\phi }\left(s_{t}\right)\right)-Q_{\theta }\left(s_{t}\right)\right]\right]$$

The subscript ϕ and θ represent the parameters of the policy neural network and the Q-function neural network separately. Training data $s_{t}$ is sampled from a replay buffer D since the maximum entropy reinforcement framework is an offline learning algorithm.

#### 2.3.3. SAC-Discrete Algorithm

SAC-Discrete uses the maximum entropy reinforcement framework to train an agent and uses a clipped double-Q trick to avoid Q-value overestimate [28]. We added a bar on top of the notation to denote a target network, and the target network smoothly updates with Polyak averaging using a hyperparameter τ. This hyperparameter τ is between 0 and 1, τ∈[0,1]. SAC-Discrete is given by Algorithm 1.
**Algorithm 1** Soft Actor–Critic with Discrete Actions Setting (SAC-Discrete).1: Initialize local networks $Q_{\theta_{1}}:S\to R^{\left|A\right|}$, $Q_{\theta_{2}}:S\to R^{\left|A\right|}$, and $\pi_{\phi }:{\left[0,1\right]}^{\left|A\right|}$2: Initialize target networks $\bar{Q_{\theta_{1}}}:S\to R^{\left|A\right|}$, and $\bar{Q_{\theta_{2}}}:S\to R^{\left|A\right|}$3: Equalize target and local network parameters $\bar{\theta_{1}}\leftarrow \theta_{1}$, and $\bar{\theta_{2}}\leftarrow \theta_{2}$4: Initialize an empty replay buffer D←∅5: **repeat**
6:  Observe state $s_{t}$ and select an action $a_{t}∼\pi_{\phi }\left(a_{t}|s_{t}\right)$7:  Execute $a_{t}$ in the environment8:  Observe next state $s_{t+1}$, reward $r_{t+1}$, and done signal $d_{t+1}$, where $s_{t+1},r_{t+1}∼p\left(s_{t+1},r_{t+1}|s_{t},a_{t}\right)$9:  Store the transition in the replay buffer $D\cup \{\left(s_{t},a_{t},r_{t+1},s_{t+1},d_{t+1}\right)\}$10:  **if** it’s time to update **then**11:   Random sample a batch of transitions $B=\{\left(s_{t},a_{t},r_{t+1},s_{t+1},d_{t+1}\right)\}\in D$12:   Compute the target soft Q-value$y\left(r_{t+1},s_{t+1},d_{t+1}\right)=r_{t+1}+\gamma \left(1-d_{t+1}\right)V\left(s_{t+1}\right)$,where $V\left(s_{t+1}\right)=\pi_{\phi }{\left(s_{t+1}\right)}^{T}\left[\min_{i=1,2}\bar{Q_{\theta_{i}}}\left(s_{t+1}\right)-\alpha \log\left(\pi_{\phi }\left(s_{t+1}\right)\right)\right]$13:   Update Q-functions by one step of gradient decent using${▽}_{\theta_{i}}\frac{1}{\left|B\right|}\sum_{(s_{t},a_{t},r_{t+1},s_{t+1},d_{t+1})\in B}{\left[Q_{\theta_{i}}\left(s_{t}\right)-y\left(r_{t+1},s_{t+1},d_{t+1}\right)\right]}^{2}$14:   Update policy by one step of gradient decent using${▽}_{\phi }\frac{1}{\left|B\right|}\sum_{s_{t}\in B}\pi_{\phi }{\left(s_{t}\right)}^{T}\left[\alpha \log\left(\pi_{\phi }\left(s_{t}\right)\right)-\min_{i=1,2}Q_{\theta_{i}}\left(s_{t}\right)\right]$15:   Update target network parameters $\bar{\theta_{i}}\leftarrow \tau \bar{\theta_{i}}+\left(1-\tau \right)\theta_{i}$ for i∈{1,2}16:  **end if**17: **until** convergence

### 2.4. Applying SAC-Discrete for the Sensor Placement Environment

#### 2.4.1. Neural Network Structure

**Figure 5:**
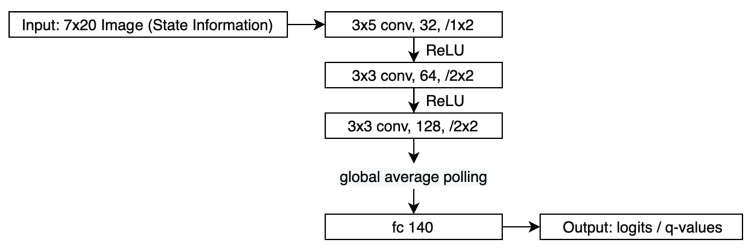
Policy and Q-function neural network structure.

To apply SAC-Discrete, we need to design the policy and soft Q-function network. The policy network and the soft Q-function network input an 7 × 20 image as the state information and output each position’s logit or Q value dependent on the network type. Since both networks share the same input and output shapes, we used the same design structure, as Figure 5 shows.

#### 2.4.2. Testing Sensor Placement Environment with Created Video

**Figure 6:**
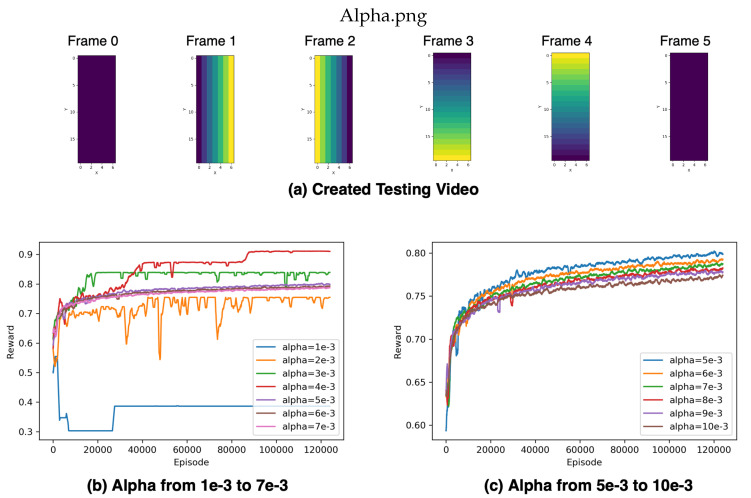
Illustration of created testing video and episodic rewards. (**a**) The first and last frames are empty images without any pressure. Using a simple increase and a decrease patterns to generate the rest frames, (**b**,**c**) are the episode reward using different temperature parameters from $1\times 10^{-3}$ to $10\times 10^{-3}$ and filtered by a moving average filter with a window size 1000.

We created a testing video with a simple pattern, as shown in Figure 6a, in order to test the sensor placement environment. Meanwhile, we set up various temperature hyperparameters for this experiment. Temperature hyperparameter affects the final training reward and the convergence time. We tested ten temperatures from $1\times 10^{-3}$ to $10\times 10^{-3}$. When the temperature is too low, like alpha equals $1\times 10^{-3}$, the agent lacks discovery and training stability and performs the worst, as shown in Figure 6b. Using a higher temperature increases training stability. However, the convergence time increases as the temperature value increases as well, as shown in Figure 6c. The result showed that selecting a proper temperature hyperparameter is critical, not only increases the training stability and final reward but also decreases the training time like alpha equals $4\times 10^{-3}$.

#### 2.4.3. Tuning Temperature with Population Based Training

To select a proper temperature hyperparameter, we utilized the Population Based Training (PBT) method [20]. This method combines the parallel search and sequential optimization hyperparameters tuning method. First, the PBT method initializes the population with some agents, which have various hyperparameters. After a training period, it exploits agents whose performance in the top 20% of the population to replace the bottom 20%. Meanwhile, perturbing the hyperparameters to explore the hyperparameter space. Keep repeating the exploit-and-explore process to tune the hyperparameters. For a population P with *N* training models ${\left\{\theta^{i}\right\}}_{i=1}^{N}$ initialized with different hyperparameters ${\left\{h^{i}\right\}}_{i=1}^{N}$, the PBT method is given by Algorithm 2.

Applying the PBT method for the sensor placement task, we created a population with a size of 15 and only allowed the PBT method to optimize the temperature parameter. The temperature parameter is initialized with a log scale uniform distribution between $1\times 10^{-1}$ to $1\times 10^{-3}$. Functions that invoked in the PBT method are described in the following:

- Step: Each training iteration updates by the gradient descent with Adam optimizer [29], the learning rate is set to $3\times 10^{-4}$.
- Eval: We evaluate the current model with averaging the last 10 episodic rewards.
- Ready: A member of the population is considered ready to go through the exploit-and-explore process when the agent elapsed $5\times 10^{5}$ agent steps since the last time that it was ready.
- Exploit: First, we rank all the members of the population using the evaluation value. If the current member is in the bottom 20% of the population, we randomly sample another agent from the top 20% of the population and copy its parameters and hyperparameters.
- Explore: We randomly perturb the hyperparameters by a factor of 0.8 or 1.2.

**Figure 7:**
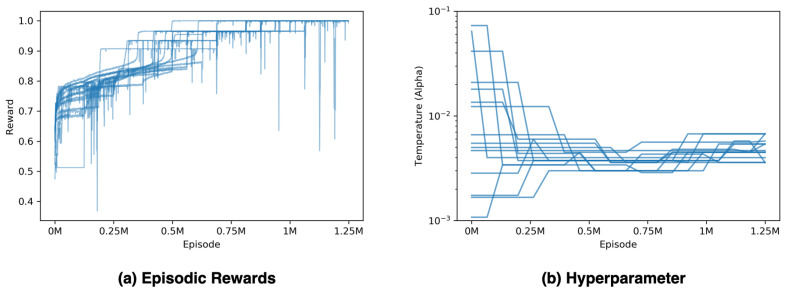
Illustration of tuning temperature hyperparameter with the PBT method. (**a**) is the episodic rewards for 15 agents and filtered with a moving average filter with a window size 1000; (**b**) displays each agent’s temperature hyperparameter with a log scaling.

The whole training process runs for 10 M agent steps, which is 1.25 M episodes since each episode takes eight agent steps until terminated. All agents have learned the optimal policy in the testing video’s sensor placement environment; as Figure 7a shows, the maximum episodic reward in the sensor placement environment that can be obtained is 1. Moreover, the PBT method adjusts a hyperparameter during a training process, as shown in Figure 7b.
**Algorithm 2** Population based training (PBT).1: Initialize population P2: **for**
(θ,h,p,t)∈P (asynchronously in parallel) **do**3:  **while** not end of training **do**4:   One step of optimization using hyperparameters *h*. θ← step(θ|h)5:   Current model evaluation. p← eval(θ)6:   **if** ready(p,t,P)
**then**7:    Use the rest of the population to find better solution. $h^{{}^{\prime }},\theta^{{}^{\prime }}\leftarrow$ exploit(h,θ,p,P)8:    **if**
$\theta \neq \theta^{{}^{\prime }}$
**then**9:     Produce new hyperparameters *h*. h,θ← explore $\left(h^{{}^{\prime }},\theta^{{}^{\prime }},P\right)$10:     New model evaluation. p← eval(θ)11:    **end if**12:   **end if**13:   Update population with new (θ,h,p,t+1)14:  **end while**15: **end for**16: Select the model with the highest *p* in P


## 3. Results

To optimize the sensor placement for the foot plantar center of pressure without any prior knowledge, we proposed the sensor placement environment and solved it with the SAC-Discrete algorithm. Using the reward redistributed trick to make the training process feasible, as mentioned in Section 2.2.3, and the PBT method to tune the temperature hyperparameter makes the training process more stable and better performing, as mentioned in Section 2.4.3. In the testing video task, this mechanism achieves the optimal sensor placement for the COP trajectory, as shown in Figure 7a; this experiment demonstrates the robustness of the training process.

**Figure 8:**
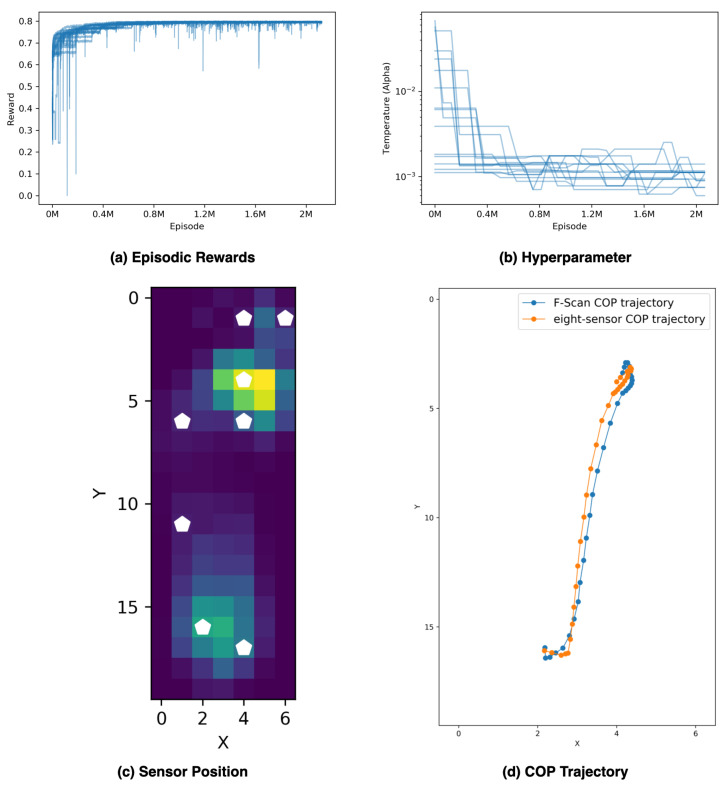
Illustrate the result of applying the sensor placement environment for self-selected speed running tasks. (**a**) is the episodic rewards for 15 agents and filtered with a moving average filter with a window size 1000; (**b**) displays each agent’s temperature hyperparameter with a log scaling; (**c**) presents the final designed sensor position from the best agent within the population, and the background is a pixel-wise accumulated stance-phase image; (**d**) shows the difference between the COP trajectory from the F-Scan System and our designed eight-sensor setting.

**Figure A1:**
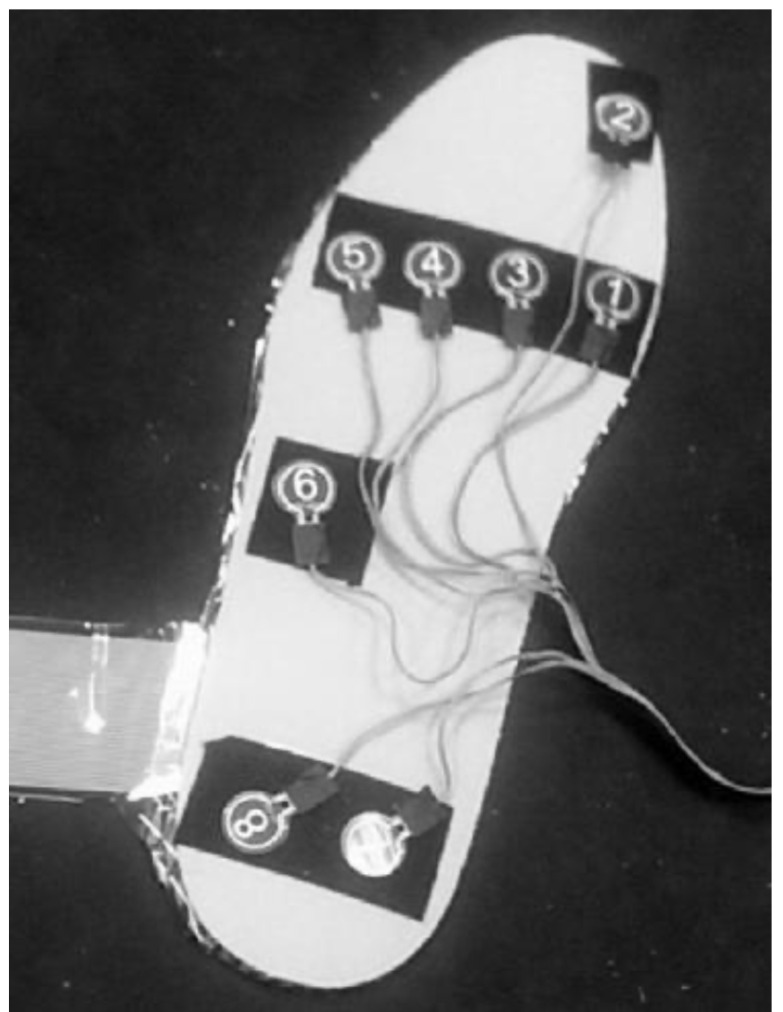
WalkinSense^®^ sensor placement.

For the self-selected speed running task, we fed 150 stance-phase plantar pressure videos to the sensor placement environment. Hyperparameters setup for SAC-Discrete in this experiment can be found in Appendix A Table A1, and the PBT setup for tuning temperature parameter can be found in Appendix B Table A2. We ran this experiment for 17 M agent steps, which is 2.125 M episodes. The best agent within the population gets an average reward of 0.7986 in the final 1000 episodes, as Figure 8a shows. Rewards start to converge around 0.8 M episodes, and so does the temperature hyperparameter, as Figure 8b shows. The final designed sensor placement position is presented in Figure 8c. The difference of the COP trajectory between the F-Scan System and the designed eight-sensor setting is shown in Figure 8d. We compared our designed eight-sensor setting with the placement design using the concept of WalkinSence [30], as Table 1 shows. Table 1 clearly shows that the performance of our method obtains a higher average reward. The sensor placement design for the WalkinSense can be found in Appendix C Figure A1.

## 4. Discussion

**Figure 9:**
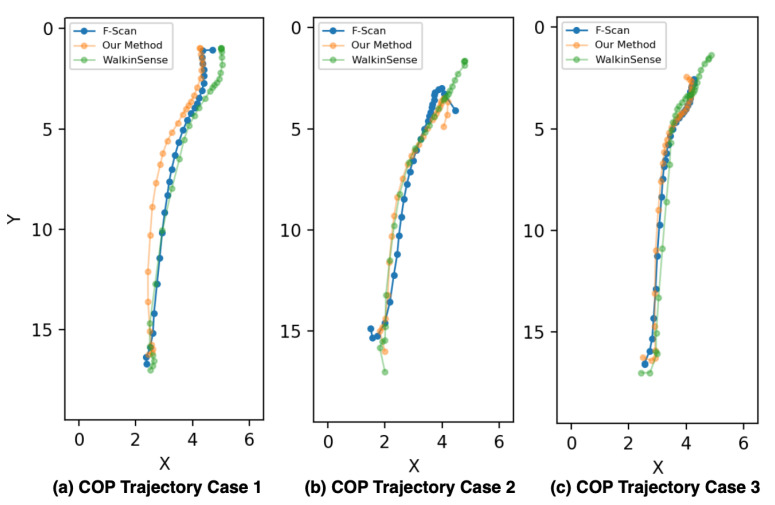
Illustrate the COP Trajectory difference between our method and WalkinSense.

Although this study proposed a method that can find a sensor placement within a large number of combinations, we only applied it for finding an eight-sensor placement for self-selected speed running tasks. Applying this method for a different task is to replace the plantar pressure video from self-selected speed running tasks to others. Since the objective of this optimization is to reduce the average distance for the COP distance for each video frame, this method is putting more effort on the region where the COP is dense, as Figure 9a shows. This is the reason why our method placed two sensors in the toe region, and it also increases the accuracy in the toe-off phase, as Figure 9b,c shows. Due to the small number of participants in this experiment, the sensor placement result may not be general enough. However, it shows that this method can be applied to more than one subject and performing better in the COP trajectory accuracy. On the other hand, applying this method for only one subject can create a personalized custom sensor placement design. Using a different number of sensor counts can be studied in the future work, by increasing or decreasing the environment’s number of sensors.

## 5. Conclusions

This paper presented a sensor placement environment, which can be applied for the SAC-Discrete, a deep RL algorithm, to find the optimal sensor position for self-selected speed running tasks without any prior knowledge of the foot anatomical area. Furthermore, this work introduced a reward redistribution trick to make the training process feasible and the PBT method to tune the temperature hyperparameter making the training process more stable and better performing. The final sensor placement, determined by the best agent, achieved 0.7986 rewards for average within the environment. In summary, the sensor placement environment can find an excellent sensor position for fitting the COP trajectory without any prior knowledge of foot anatomical area, and the performance surpassed the human-designed sensor placement.

## Figures and Tables

**Table 1:** Comparison of the different sensor placements.

	WalkinSense^®^ [30]	Our Method
Average of 1000 episodic rewards	0.7117	0.7986

## Appendix A. Sac-Discrete Hyperparameters

**Table A1:** Hyperparameters used for SAC-Discrete.

Hyperparameter	Value
Adam learning rate	$3\times 10^{-4}$
Replay buffer size	$1\times 10^{6}$
Minibatch size	64
Discount (γ)	0.99
Polyak (τ)	0.997
Steps per learning update	4
Learning iterations per round	1
Initial random steps	$2\times 10^{4}$

## Appendix B. Pbt Hyperparameters

**Table A2:** Hyperparameters used for PBT.

Hyperparameter	Value
Temperature (α)	$\left[10^{-1},10^{-3}\right]$ (log scale random uniform)
Population size	15
Number of agent step for ready	$5\times 10^{5}$
Perturbing factor	0.8 or 1.2

## References

[1] Bonato P. (2003). Wearable sensors/systems and their impact on biomedical engineering. IEEE Eng. Med. Biol. Mag..

[2] Nilpanapan T., Kerdcharoen T. Social data shoes for gait monitoring of elderly people in smart home. Proceedings of the 2016 9th Biomedical Engineering International Conference (BMEiCON).

[3] Yamakawa T., Taniguchi K., Asari K., Kobashi S., Hata Y. Biometric personal identification based on gait pattern using both feet pressure change. Proceedings of the 2010 World Automation Congress.

[4] Razak A., Hadi A., Zayegh A., Begg R.K., Wahab Y. (2012). Foot plantar pressure measurement system: A review. Sensors.

[5] Mnih V., Kavukcuoglu K., Silver D., Graves A., Antonoglou I., Wierstra D., Riedmiller M. (2013). Playing atari with deep reinforcement learning. arXiv.

[6] Mnih V., Kavukcuoglu K., Silver D., Rusu A.A., Veness J., Bellemare M.G., Graves A., Riedmiller M., Fidjeland A.K., Ostrovski G. (2015). Human-level control through deep reinforcement learning. Nature.

[7] Russell S., Norvig P. (2002). Artificial Intelligence: A Modern Approach.

[8] LeCun Y., Bengio Y., Hinton G. (2015). Deep learning. Nature.

[9] Krizhevsky A., Sutskever I., Hinton G.E. Imagenet classification with deep convolutional neural networks. Proceedings of the Advances in Neural Information Processing Systems.

[10] He K., Zhang X., Ren S., Sun J. Deep residual learning for image recognition. Proceedings of the IEEE Conference on Computer Vision and Pattern Recognition.

[11] Gatys L.A., Ecker A.S., Bethge M. Image style transfer using convolutional neural networks. Proceedings of the IEEE Conference on Computer Vision and Pattern Recognition.

[12] Long J., Shelhamer E., Darrell T. Fully convolutional networks for semantic segmentation. Proceedings of the IEEE Conference on Computer Vision and Pattern Recognition.

[13] Graves A., Mohamed A.r., Hinton G. Speech recognition with deep recurrent neural networks. Proceedings of the 2013 IEEE International Conference on Acoustics, Speech and Signal Processing.

[14] Vaswani A., Shazeer N., Parmar N., Uszkoreit J., Jones L., Gomez A.N., Kaiser Ł., Polosukhin I. Attention is all you need. Proceedings of the Advances in Neural Information Processing Systems.

[15] Tsitsiklis J.N., Van Roy B. Analysis of temporal-diffference learning with function approximation. Proceedings of the Advances in Neural Information Processing Systems.

[16] Silver D., Schrittwieser J., Simonyan K., Antonoglou I., Huang A., Guez A., Hubert T., Baker L., Lai M., Bolton A. (2017). Mastering the game of go without human knowledge. Nature.

[17] Berner C., Brockman G., Chan B., Cheung V., Dębiak P., Dennison C., Farhi D., Fischer Q., Hashme S., Hesse C. (2019). Dota 2 with large scale deep reinforcement learning. arXiv.

[18] Christodoulou P. (2019). Soft actor–critic for discrete action settings. arXiv.

[19] Haarnoja T., Zhou A., Abbeel P., Levine S. (2018). Soft actor–critic: Off-policy maximum entropy deep reinforcement learning with a stochastic actor. arXiv.

[20] Jaderberg M., Dalibard V., Osindero S., Czarnecki W.M., Donahue J., Razavi A., Vinyals O., Green T., Dunning I., Simonyan K. (2017). Population based training of neural networks. arXiv.

[21] Luo Z.P., Berglund L.J., An K.N. (1998). Validation of F-Scan pressure sensor system: A technical note. J. Rehabil. Res. Dev..

[22] Putnam W., Knapp R.B. (1996). Input/data acquisition system design for human computer interfacing. Online Course Notes.

[23] Arjona-Medina J.A., Gillhofer M., Widrich M., Unterthiner T., Brandstetter J., Hochreiter S. Rudder: Return decomposition for delayed rewards. Proceedings of the Advances in Neural Information Processing Systems.

[24] Sutskever I., Vinyals O., Le Q.V. Sequence to sequence learning with neural networks. Proceedings of the Advances in Neural Information Processing Systems.

[25] Mnih V., Badia A.P., Mirza M., Graves A., Lillicrap T., Harley T., Silver D., Kavukcuoglu K. Asynchronous methods for deep reinforcement learning. Proceedings of the International Conference on Machine Learning.

[26] Schulman J., Wolski F., Dhariwal P., Radford A., Klimov O. (2017). Proximal policy optimization algorithms. arXiv.

[27] Sutton R.S., McAllester D.A., Singh S.P., Mansour Y. (2000). Policy gradient methods for reinforcement learning with function approximation. Advances in Neural Information Processing Systems.

[28] Fujimoto S., van Hoof H., Meger D. (2018). Addressing Function Approximation Error in Actor–Critic Methods.

[29] Kingma D.P., Ba J. (2014). Adam: A method for stochastic optimization. arXiv.

[30] Healy A., Burgess-Walker P., Naemi R., Chockalingam N. (2012). Repeatability of WalkinSense^®^ in shoe pressure measurement system: A preliminary study. Foot.

